# Comparative preclinical evaluation of AS01 versus other Adjuvant Systems in a candidate herpes zoster glycoprotein E subunit vaccine 

**DOI:** 10.1080/21645515.2016.1154247

**Published:** 2016-03-02

**Authors:** Michel Fochesato, Najoua Dendouga, Mathieu Boxus

**Affiliations:** GSK Vaccines; Rue de l'Institut; 89; 1330; Rixensart; Belgium

**Keywords:** herpes zoster Adjuvant System, MPL, QS-21, Varicella-zoster virus, vaccine

## Abstract

The candidate vaccine HZ/su is being developed to prevent herpes-zoster disease (HZ). HZ occurrence is attributed to declines in varicella-zoster virus (VZV) specific T-cell immunity. HZ/su contains VZV antigen, gE, and Adjuvant System AS01_B_ (liposome-based formulation of MPL and QS-21). In clinical trials, AS01_B_ enhances CD4^+^ T-cell responses to gE. In clinical trials of other vaccines, Adjuvant Systems AS03 and AS04 also enhance antigen-specific CD4^+^ T-cell responses. Hence the purpose of this study was to evaluate gE formulated with AS01_B_, AS01_E_ (50% less MPL and QS-21 than AS01_B_), AS03 or AS04 in C57BL6 mice primed with live-attenuated VZV. Four-weeks post-vaccination, the gE-specific CD4^+^ T-cell response to gE/AS01_B_ was 5.4, 2.8 and 2.2-fold greater than those to gE/AS03, gE/AS04 and gE/AS03, respectively (*p*<0.001). Therefore in the VZV-primed mouse model, CD4^+^ T-cell responses to gE appeared most enhanced by AS01_B_, and adds further support for the use of AS01_B_ in the HZ/su formulation.

Herpes zoster (HZ) or shingles is a disease with symptoms including skin rash and postherpetic neuralgia, and is caused by the reactivation of dormant varicella zoster virus (VZV).^1^ The lifetime risk of having HZ has been estimated at around 30%, and the incidence of disease increases with age, with immunosuppressive treatments or with immunocompromised conditions.^1-3^ The occurrence of HZ has been attributed to a decline in T-cell mediated immunity to VZV.^1,2,4,5^ A live-attenuated vaccine is licensed (*Zostavax*^®a^, Merck),^3,6,7^ and its effectiveness against the incidence of disease has been estimated at around 50% in adults aged ≥60 years.^6,8^ Therefore there remains a need to increase vaccine effectiveness, especially in older adults.

In a recent Phase 3 placebo-controlled study of the candidate subunit vaccine HZ/su, the efficacy for the prevention of HZ was estimated at 97.2% in adults ≥50 years of age; and vaccine efficacy in the ≥70 years age group was similar to that in the 50–59 y and 60–69 y age groups.^9^ HZ/su includes a recombinant antigen based on the VZV glycoprotein E (gE). VZV gE is an abundant virion-envelope glycoprotein, essential for viral replication and cell-to-cell spreading;^10,11^ and gE-specific CD4^+^ T cells may play a role in preventing symptomatic VZV reactivation in healthy adults.^5,12^

HZ/su also contains the GSK-proprietary Adjuvant System AS01_B_, which enhances both gE-specific antibody and T-cell responses to antigen in mice and in clinical trials.^13-17^ AS01_B_ contains liposomes and 2 immunostimulants, MPL (3-*O*-desacyl-4'-monophosphoryl lipid A) and QS-21^d^ (*Quillaja saponaria* Molina, fraction 21).^17^ In mice, MPL and QS-21, in combination, synergistically induce gE-specific CD4^+^ T-cell responses to vaccination.^13^ AS01_B_ contains 2-fold more MPL and QS-21 than AS01_E_, and induces higher gE-specific CD4^+^ T-cell responses to vaccination than AS01_E_ in mice and in a clinical trial of human adults aged ≥50 years.^13,15^ AS01_B_ also induces higher gE-specific CD4^+^ T-cell responses to vaccination in mice than an aluminum-salt adjuvant.^13^

In clinical trials, 2 other Adjuvant Systems, AS03 and AS04, have been shown to enhance antigen-specific CD4^+^ T-cell responses to influenza and human papillomavirus HPV vaccines, respectively, but have not been evaluated with the gE antigen.^18,19^ Therefore the objective of this study was to compare CD4^+^ T-cell responses to gE vaccines adjuvanted with AS01_B_ or AS01_E_, with those to gE vaccines adjuvanted with AS03 or AS04, in mice primed with live-attenuated VZV. Antibody responses to vaccination were also evaluated.

Two independent experiments were performed in which C57Bl/6 mice (Harlan Horst, Netherlands) were primed with one sub-cutaneous dose of a live-attenuated varicella vaccine (full-human dose of *Varilrix*^®bc^; 10^4^ pfu). Five weeks after priming on Days 0 and 28, mice were administered intramuscular (*tibialis*) injections of a gE vaccine^c^ or saline (0.9% NaCl; control group). One gE-vaccine dose contained 5 µg gE and an Adjuvant System in 50 µl. The contents of the Adjuvant Systems AS01_B_, AS01_E_, AS03, or AS04 are defined by the quantities of the following components in a full-human dose: AS01_B_ contains 50 µg MPL and 50 µg QS-21; AS01_E_ contains 25 µg MPL and 25 µg QS-21; AS03 contains 11.86 mg α-tocopherol and squalene in an oil-in-water emulsion, and AS04 contains 50 μg MPL adsorbed on 500 μg aluminum salt. For the purpose of this article, gE/AS01_B_, gE/AS01_E_, gE/AS03, and gE/AS04 refer to the mouse vaccines in which the Adjuvant System contains one tenth of the respective quantities used in a full-human dose.

Antigen-specific CD4^+^ T cells expressing at least one of the 2 cytokines IFN-γ and IL-2, were detected in all vaccine groups at 30 d after dosing. In Experiment 1, the geometric mean frequency (GMF) of gE-specific CD4^+^ T cells was 6.2% in the gE/AS01_B_ group; whereas it was 3.5% in the gE/AS04 group, 2.2% in the gE/AS01_E_ group and 1.3% in the gE/AS03 group (Fig. 1A). In Experiment 2, the GMF of gE-specific CD4^+^ T cells was 9.1% in the gE/AS01_B_ group; whereas it was 5.8% in the gE/AS01_E_ group, 1.9% in the gE/AS04 group, and 1.5% in the gE/AS03 group (Fig. 1A). In Experiments 1 and 2, the frequencies of gE-specific CD4^+^ T cells in the NaCl group were either close to or below the cut-off for the assay (GMFs were 0.3% and 0.05%, respectively). The frequencies of gE-specific CD8^+^ T cells in any of the adjuvanted-vaccine groups were not significantly higher than the baseline frequencies observed in the NaCl group (not shown).

**Figure 1.**
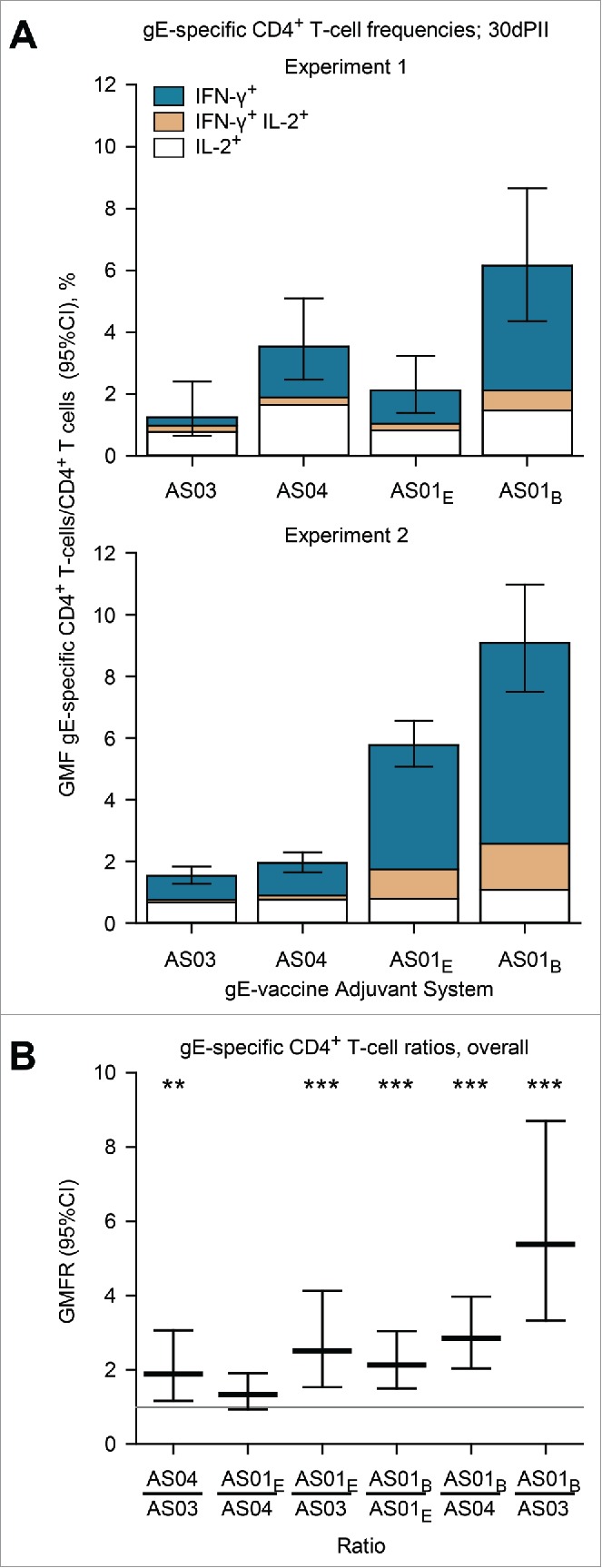
Geometric mean frequencies (GMFs) of (A) gE-specific CD4^+^ T cells and (B) ratios of GMFs from different adjuvanted-vaccine groups. Spleens (Experiment 1, N=8 and Experiment 2, N = 11; spleens pooled from 2 mice) were sampled at 30 d after the second vaccine dose (30dPII). The frequency of gE-specific CD4^+^ T cells was calculated as a percentage of cytokine-positive CD4^+^ T cells divided by all CD4^+^ T cells. Error bars represent 95% confidence intervals. In Experiments 1 and 2, the frequencies of antigen-specific CD4^+^ T cells in the NaCl group were either close to or below the cut-off for the assay (GMFs were 0.3% and 0.05%, respectively). In (B), horizontal gray reference line indicates a ratio = 1, and asterisks indicate significant differences from 1 (** *p*<0.01; *** *p*<0.001). Antigen-specific T cells were evaluated in splenocyte-restimulation cultures as described previously,^13^ but with some modifications. Briefly, splenocyte cultures (110^6^ cells per well of 96-well plate) were prepared from spleens of 2 mice and were incubated for 2 hours in the presence of gE peptides spanning the complete gE protein (6315-mer peptides, 11 amino-acid overlap) and then incubated ˜18 hours in the presence of brefeldin A. Subsequently, the cells were stained with fluorescent-monoclonal antibodies specific for CD4 and after permeabilization, for intracellular-cytokines IL-2 and IFN-γ. All antibodies were obtained from BD Biosciences, Belgium. Flow cytometry was performed using LSR II Facs (BD Biosciences, Belgium) and analyzed using FlowJo software (FlowJo, LLC, OR, USA). Statistical calculations were based on an analysis of variance with 2 factors (vaccine group, experiment) on log_10_ values using a heterogeneous variance model (i.e., identical variances were not assumed for the different levels of the factor). Estimates of the geometric-mean ratios between groups and their 95% confidence intervals (CI) were obtained using back-transformation of log_10_ values. Adjustments for multiple testing were performed using Tukey's method. All analyses were performed using SAS software (Version 9.2, SAS Institute Inc., NC, USA).

In both experiments, the differences between the vaccine groups were mostly associated with gE-specific CD4^+^ T cells that were IFN-γ positive (Fig. 1A). Moreover, the magnitude of IFN-γ production in IFN-γ^+^ CD4^+^ T cells relative to IFN-γ^−^ CD4^+^ T cells appeared higher in the gE/AS01_B_ group than in the other groups (measured by fluorescent-staining intensities; not shown). Overall, gE-specific CD4^+^T cells were 5.4, 2.8 and 2.2-fold more frequent in response to gE/AS01_B_ than in response to gE/AS03 gE/AS04 and gE/AS01_E_ (p<0.001), respectively; and were 2.5-fold more frequent in response to gE/AS01_E_ than in response to gE/AS03 (p<0.001; Fig. 1B).

Antigen-specific antibodies were detected in all vaccine groups at 14 and 28 d after dosing but were not detected in the NaCl group (concentrations were below the cut-off of the assay; i.e. <500 EU/ml). For both experiments and in any given vaccine group, the magnitude of geometric mean concentrations (GMCs) of gE-specific antibodies appeared similar at 14 d compared with 28 d (Fig. 2A). Some significant differences were observed in the ratios of antibody concentrations between vaccine groups, although, no differences were more than 2-fold (Fig. 2B). In the gE/AS01_B_ group at 14 and 28 days, GMCs were 1.6-fold (*p*<0.001) and 1.7-fold higher (*p*<0.001) than in the gE/AS03 group, respectively; 1.4-fold (*p*<0.001) and 1.7-fold (p<0.001) higher than in the gE/AS04 group, respectively; and 1.5-fold (*p*<0.001) and 1.4-fold (p<0.05) higher than in the gE/AS01_E_ group, respectively (*p*<0.001).

**Figure 2.**
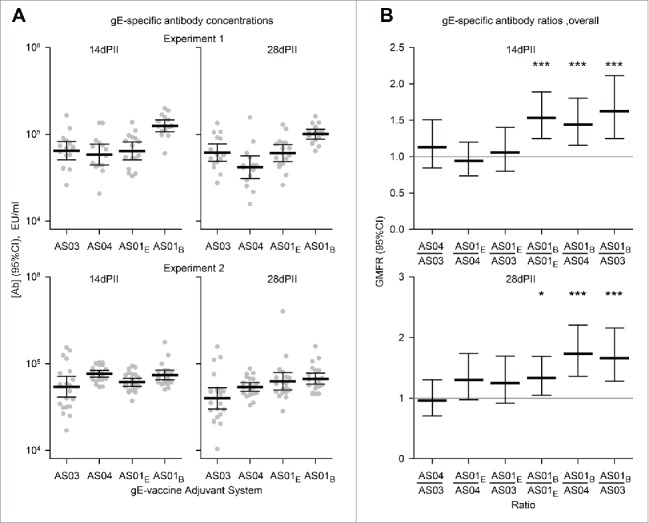
Geometric mean concentrations (GMCs) of (A) gE-specific antibodies and (B) ratios of GMCs (GMFRs) from different adjuvanted-vaccine groups. Sera (Experiment 1, N = 16 1 and Experiment 2, N = 22) were sampled at 14 and 28 d after the second vaccine dose (14dPII and 28dPII, respectively). Error bars represent 95% confidence intervals. Antigen-specific antibodies were not detected in the NaCl group (concentrations were below the cut-off of the assay; i.e.<500 EU/ml). In (B), horizontal gray reference lines indicate a ratio = 1, and asterisks indicate significant differences from 1 (*p<0.05; *** *p*<0.001). Antigen-specific antibody concentrations (in EU/ml and defined by internal standards) were measured by ELISA as previously described.^13^ Statistical calculations were performed as described in Figure 1.

Overall, the AS01_B_-based vaccine formulation induced the highest frequency of gE-specific CD4^+^ T cells compared with the AS03 and AS04 vaccine formulations, primarily reflecting differences in the frequencies of those T cells that were IFN-γ positive. The AS01_E_ formulation also induced a higher frequency of gE-specific CD4^+^ T cells than AS03. The potential that these comparative differences are relevant in humans is suggested from the observation that gE-specific CD4^+^ T cell responses were higher to the AS01_B_ formulation than to the AS01_E_ formulation in VZV-primed mice (consistent with a previous study) as well as in the clinical setting.^13,15^

Although VZV antibodies are not considered essential to confer protection against HZ,^2^ the AS01_B_-based vaccine formulation induced marginally higher gE-specific antibody concentrations compared with the other formulations (and our unpublished observations suggest that these antibody concentrations correlate with VZV-neutralizing activity). Hence, the differences between AS01_B_-based vaccine formulation and the AS03- and AS04-based vaccine formulations were primarily reflected in differences in CD4^+^ T cell frequencies and in line with nonclinical and clinical experience of other vaccines.^17^

In humans, VZV-specific cell-mediated immunity appears to play an essential role in protection against both the occurrence and morbidity of HZ, although a clearly defined correlate of protection against HZ remains to be identified.^1,2,4,5^ CD4^+^ T cells expressing IFN-γ appear to predominate in the responses to VZV antigens in general or to gE alone,^5,20^ thus supporting the monitoring of CD4^+^ T cells in HZ-vaccine evaluations.^13^ Hence the present study and a previous preclinical study add further support for the use of AS01_B_ rather than AS03, AS04, AS01_E_ or aluminum salt in the candidate HZ vaccine formulation.

## Abbreviations

CI, confidence interval;
gE, glycoprotein E;
HZ, herpes zoster;
GMC, geometric mean concentration;
GMF, geometric mean frequency;
MPL, 3-O-desacyl-4'-monophosphoryl lipid A;
QS-21, Quillaja saponaria Molina, fraction 21;
VZV, varicella zoster virus

## Abbreviations

*CI*: confidence interval
*gE*: glycoprotein E
*HZ*: herpes zoster
*GMC*: geometric mean concentration
*GMF*: geometric mean frequency
*MPL*: 3-O-desacyl-4'-monophosphoryl lipid A
*QS-21*: Quillaja saponaria Molina, fraction 21
*VZV*: varicella zoster virus
